# Effect of In Situ Polymerization of Super Absorbent Polymers on the Protection of Earthen Heritage Sites in Semi-Arid Regions

**DOI:** 10.3390/ma17194839

**Published:** 2024-09-30

**Authors:** Yao Zheng, Senbiao Liu, Yifan Zhang, Zhilin Du, Miao Li, Hongjie Luo, Jianfeng Zhu

**Affiliations:** 1 Shaanxi Key Laboratory of Green Preparation and Functionalization for Inorganic Materials, Institute of Silicate Cultural Heritage, School of Material Science and Engineering, Shaanxi University of Science and Technology, Xi’an 710021, China; 220212082@sust.edu.cn (Y.Z.); 210212092@sust.edu.cn (S.L.); 220212158@sust.edu.cn (Y.Z.); 210211004@sust.edu.cn (Z.D.); 18792418608@163.com (M.L.); 2 Research Institute of Cultural Relics, Shanghai University, Shanghai 200444, China; hongjieluo@shu.edu.cn

**Keywords:** super absorbent polymer, earthen sites, soil water retention, heritage conservation

## Abstract

As precious cultural heritage, earthen sites are susceptible to various natural factors, leading to diverse forms of degradation. To protect earthen sites, the effects of super absorbent polymers (SAPs) on soil water retention, physical properties, and color compatibility at different concentrations were studied. After applying SAP treatment to an earthen site with different degrees of weathering, we drew the following conclusions. SAP-2 improved soil water retention capacity, increased soil water content, and slowed down the precipitation of soluble salts. At the same time, SAP-2 had the least effect on soil color difference and reduced the development of cracks by filling soil pores and enhancing the cohesion between soil particles, thus giving the earthen sites better weathering resistance. Therefore, the results provide a useful reference for the surface cracking of earthen sites in semi-arid areas and the degradation caused by flaking and block spalling.

## 1. Introduction

Earthen sites are left over from ancient human activities and have important historical and cultural value [1]. They are a witness to human civilization and an important record of ancient society and culture. Understanding the development and changes in ancient society and culture is significant. Earthen sites are widely distributed in arid and semi-arid areas in northwest China. Most of these earthen sites are exposed to the natural environment. The sites have become fragile after thousands of years of sand and rain erosion, and much degradation exists. Therefore, anti-weathering reinforcement is the main direction of the protection of earthen sites in the northwest region, and soil stability is a crucial factor for reinforcement. To evaluate the stability of earthen site slopes, Nikolaos [2] used RUSLE technology to simulate soil erosion after fire by combining soil monitoring with numerical simulation, thus providing useful insights for soil erosion estimation and identification of high-risk erosion areas. Numerical simulations have also been applied to analyze the cyclic expansion and contraction characteristics of loess slopes, such as the Jiaohe site in China [3]. Numerical simulation technology can simulate and analyze various factors affecting the stability and erosion of earthen site slopes and provide a scientific basis and guidance for the formulation of targeted reinforcement and protection measures.

Because of the semi-arid climate in the northwest region, the surfaces of earthen sites are dry all year round, and the influence of wind erosion aggravates surface deterioration. The surface erosion caused by wind is one of the most important factors contributing to the deterioration of earthen sites in northwest China [4] as it threatens their stability and complicates protection work. The protection of earthen sites includes desalination, grouting [5,6], reinforcement, and surface protection, among which research on reinforcement is the most extensive. Chai et al. [7] used methacrylic acid resin to reinforce and protect Tianluo Mountain earthen sites. Their results showed that methacrylic acid resin can effectively improve the strength of earthen sites. Ethyl orthosilicate with good permeability has also attracted the attention of scholars. The color difference in reinforced earthen sites is small, and the mechanical properties and durability are also significantly improved [8]. By adding different contents of nano-SiO_2_ to loess to reinforce the soil, Kong et al. [9] studied the changes in microstructure and mineral composition of solidified loess under different curing times. The mechanical test results showed that the mechanical properties of loess were directly proportional to the content of nano-SiO_2_ and curing days. Zhang et al. [10] used a 1% concentration of SH (modified polyvinyl alcohol) to reinforce earthen sites. Their results showed that the mechanical properties, wind erosion resistance, rain erosion resistance, and salt erosion resistance of the reinforced soil were significantly improved. Lanzón, Victoria et al. [11,12] found that CO_2_ in the air can be carbonized on the surface of the soil to form a nano-CaCO_3_ coating by using the characteristics of nano-Ca(OH)_2_. The coating can consolidate soil pores and hinder water infiltration into the soil, improving the durability of earthen sites. In addition, silicone materials [13,14] and acrylic resins [15,16] are used for sandstone and earthen site protection because of their good permeability and adhesion. Obviously, these anti-weathering reinforcements protect earthen sites by increasing the mechanical properties of the soil to a certain extent.

Suppose that water evaporation can be slowed and earthen sites' water-holding capacities can be increased. In that case, soil moisture can be effectively maintained, and the erosion of earthen sites by wind can be reduced. Hydrogel has been widely used in the field of agriculture. It has a three-dimensional network structure. After absorbing water, it expands and retains a large amount of water. When ambient humidity decreases, it can slowly release water and increase soil moisture content. After the gel is combined with soil, its expansion shrinkage process is conducive to controlling the loss caused by water absorption and soil release. It can change the pore distribution in the soil, adjust soil moisture [17], and enhance soil agglomeration and water retention capacity [18]. In particular, SAP hydrogel can improve sandy soil [19]. An appropriate amount of SAP can improve the water retention capacity of sandy soil so as to maximize its water retention [20]. In arid areas, SAP gel is uniformly applied to soil before rainfall, and it can significantly reduce surface runoff and soil erosion [21,22]. If applied to earthen sites in semi-arid areas, it can increase the water content of earthen sites, increase the water-holding capacity of the soil, and reduce soil cracking. Moreover, under the premise of compliance with the industry standards of NY/T 886-2022 [23] and evaluation requirements of NY/T 2271-2016 [24], the application of SAP as a soil amendment is deemed safe. Its positive effects on soil quality and promotion of crop growth in agricultural and environmental engineering applications, particularly in arid and semi-arid regions, have been demonstrated. Although SAP colloid has excellent performance in soil water retention, it must be mixed with soil to protect earthen sites, which is bound to cause damage to them.

Based on this background, in this experiment, an in situ self-cross-linked SAP developed in a laboratory was used for the on-site protection of a site in Shaanxi. By evaluating the effects of different concentrations of SAP reinforcement on the water retention performance and morphological changes at other earthen sites, the optimal SAP concentration suitable for the earthen sites in this area was selected. The test results can provide a reference for protecting earthen sites under similar climatic conditions.

## 2. Characteristics of Geological Conditions

### 2.1. Deterioration Characteristics

**Figure 1:**
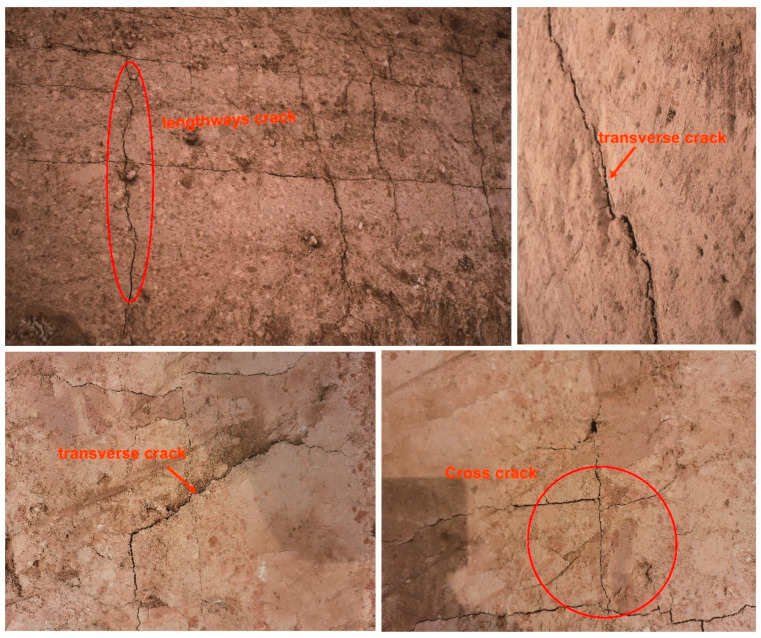
Typical crack deterioration at the site.

The experimental site is near Lishan Mountain in Lintong, a “Ya”-shaped tomb. The tomb chamber is a huge square or “Ya”-shaped vertical earthen pit with a tomb passage on each side. The overall shape is similar to the “Ya” character in ancient Chinese script. It is one of the tomb forms of ancient Chinese aristocrats or emperors. The GPS coordinates of the site are 34° 32′ N and 109° 14′ E; it is identified as belonging to the tomb of an emperor who ruled the central plains of ancient China, dating back 2000 years. Because of its great value and grand scale, this tomb has been excavated since 2018, and it is of great significance to the study of the development and changes in the history of ancient Qin in China. The deterioration mainly results in fissures, gullies, denudation, collapse, crisp powder, and so on, as observed through on-site investigation. Fissures are a common form of deterioration in earthen sites. Many factors cause them. If the site is not protected, gullies due to rain can easily develop and even cause the site to collapse. Example cracks at the site, mainly longitudinal and oblique, are shown in Figure 1.

**Figure 2:**
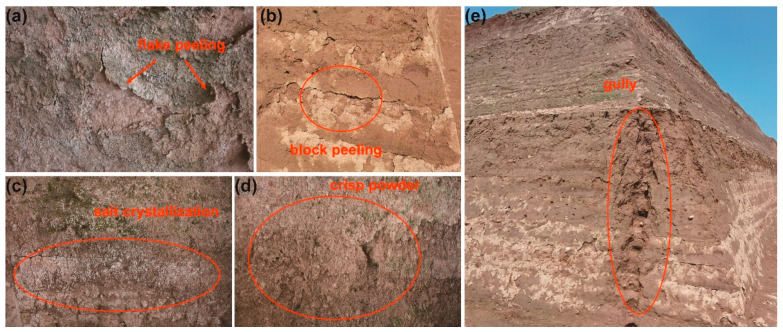
Typical deterioration at the site. (**a**) Flake peeling, (**b**) block peeling, (**c**) salt crystallization, (**d**) crisp powder, and (**e**) a gully.

Because of its location in the transition zone between the mild temperate semi-humid climate of East Asia and the inland desert climate, this place exhibits the features of both climatic types. During the summer, frequent thunderstorms are characterized by significant amounts of precipitation within a brief duration. Furthermore, the site has a substantial quantity of fissures. Because of the ongoing erosion caused by precipitation, several tiny gullies are created at the highest point of the earthen area, and these fractures progressively widen to develop into fissure gullies. The more giant broken gully head has a slender and profound morphology. It traverses the complete platform profile (Figure 2e).

Sustained development will lead to the collapse and significant degradation of the original state of the earthen site. The site is experiencing significant surface erosion, primarily characterized by the detachment of flakes and blocks, as seen in Figure 2a,b. Lin et al. [25] examined the surface spalling pattern and development characteristics of the site and determined that the spalling pattern mainly consisted of muddy spalling and salt spalling. Furthermore, the earthen sites on the third level exhibit a significant whitening effect due to the presence of soluble salt, resulting in a heavily powdered surface (Figure 2c,d).

### 2.2. Basic Properties of the Site’s Soil

#### 2.2.1. Basic Physical Properties of Soil

To prevent the degradation of the earthen sites while sampling, the collapse site location was chosen to obtain the soil sample. Before sampling, the top layer of soil, about 10 cm deep, was excavated from the collapse site. Subsequently, the soil sample was carefully sealed and kept on-site to maintain its natural condition. Based on the geotechnical testing procedures outlined in Chinese regulations [26], the natural moisture content of soil samples was determined by subjecting them to the drying process at a temperature of 105 °C for 12 h. The wax sealing technique was used to assess the natural density. The dry density of soil samples was determined based on the experimental data of the soil's natural moisture content and density. Soil samples smaller than Φ5 mm × 5 mm were chosen and subjected to drying using a fully automated mercury intrusion porosimeter (MIP, Autopore IV 9500, Micromeritics, USA) to measure soil porosity and other relevant information.

The liquid and plastic limit of soil samples were measured using the liquid and plastic limit combination tester (GYS-2), and the plastic index (Ip) of the soil samples was calculated as the difference between the liquid and plastic limits. The fundamental physical characteristics of the soil at the location were measured, as shown in Table 1.

#### 2.2.2. Elemental Composition of Soil

The elemental composition of the site soil was analyzed using an energy-dispersive X-ray fluorescence spectrometer (XRF, XTG-7200V, HORIBA Scientific, Japan). The desiccated and pulverized soil samples were compressed into round discs using a tablet machine. Before the test, copper plates were used to calibrate the equipment. The dominant elements in the site soil, as shown in Table 2, are silicon (Si), aluminum (Al), and calcium (Ca).

#### 2.2.3. Soluble Salt Analysis

An ion chromatograph (Diane, ICS-900, Thermo Fisher Scientific, USA) was used to assess the soluble salt concentration in the soil. The soil samples were collected, dried, crushed, and processed using a soil–water ratio of 1:5. The samples were subjected to centrifugation at 8000 revolutions per minute for 10 min, followed by ultrasonic treatment for 0.5 h. Table 3 shows that the predominant cations in the soil of the site are Na^+^ and Ca^2+^, whereas the primary anions are SO_4_^2−^. This suggests that the damage to the earthen site is caused mainly by sulfate.

### 2.3. Experimental Area Selection

**Figure 3:**
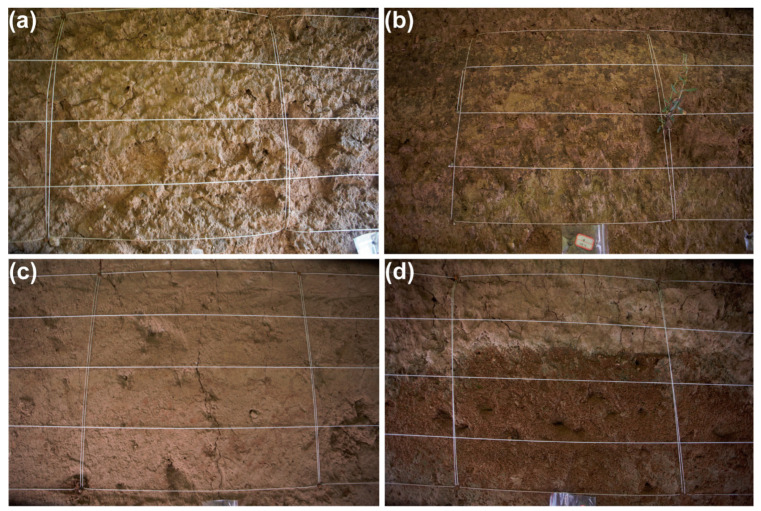
Types of experimental area selection. (**a**) Block spalling. (**b**) Sheet stripping. (**c**) Fissure. (**d**) Fragile crumble.

During the geological condition examination, it was discovered that the broad size of the site resulted in variations in the position, orientation, and water content of distinct layer profiles. Additionally, the degradation development characteristics of these profiles also varied. This study chose the test region for local reinforcement based on the intricate conditions of the site, taking into account the typical deteriorating characteristics of the earthen sites and also considering factors such as light and height. The variation in height among the first, second, and third layers of the site section leads to differences in wind force, weathering intensity, tomb illumination duration, and water content. Consequently, cracks, erosion, and other forms of damage occur. Following this concept, four representative profiles were chosen, and a test area of 200 cm in length and 50 cm in breadth was designated on the selected profile. The test space was partitioned into four test blocks, each measuring 50 cm × 50 cm. The distinctive characteristics of the reinforced region are as follows:

Type 1 refers to a significant erosion region resulting from weathering. It is situated on the ground level of every tomb route, as seen in Figure 3a.

The excavation time of the first platform is the earliest, the wind and rain erosion time exposed to the external environment is the longest, and the surface water content is in the range of 2.1~4.7%.

Type 2 is a mud-like flake peeling area caused by typical heavy rainfall, located on the second floor of the west side of the north tomb passage, as shown in Figure 3b. The moisture content is in the range of 9.8~11.3%.

Type 3 is a high water content area with small cracks on the surface, located in the third platform, and exposed to the external environment for a relatively short time, as shown in Figure 3c.

Type 4 is the serious area of surface crisp powder under the repeated dissolution–crystallization of soluble salt, as shown in Figure 3d.

## 3. Experimental Materials and Methods

### 3.1. Experimental Materials

Acrylic acid (AA), Acrylamide (AM), 2-Acrylamido-2-methyl-1-propane sulfonic acid (AMPS), and sodium thiosulfate were acquired from Shanghai Aladdin Biochemical Technology Co., Ltd. (Shanghai, China). The compound N′N′-Methylenebisacrylamide was acquired from Tianjin Kemiou Chemical Reagent Co., Ltd. (Tianjin, China). Sodium hydroxide (NaOH) was obtained from the Damao chemical reagent plant and was of analytical grade (Tianjin, China). Ammonium persulfate was acquired from Chengdu Chron Chemicals Co., Ltd. (Chengdu, China).

### 3.2. Formulation Selection

**Figure 4:**
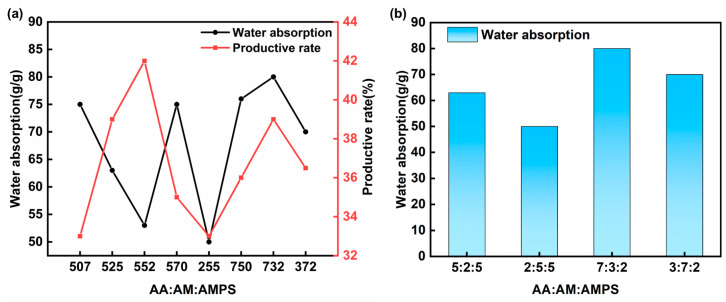
(**a**) Water absorption and yield of SAP with different ratios. (**b**) Water absorption of four different ratios of SAP.

**Figure 5:**
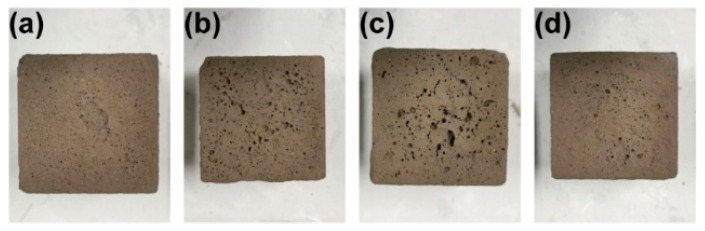
Simulated surface morphology of the sample: (**a**) 7:3:2, (**b**) 5:2:5, (**c**) 3:7:2, and (**d**) 2:5:5.

Given the varying physical and chemical characteristics of soil in different regions, it is necessary to modify the monomer ratio of the SAP formula to align with the specific soil properties. The selection of the optimal SAP monomer ratio should be based on the principle of minimizing any negative impact on the surface morphology of the soil. The experiment used dirt collected during this study to create a simulation sample of 5 cm × 5 cm × 5 cm. This sample was used to replicate the density and water content of the rammed earth layer. To preserve the surface morphology of the earthen sites after adding the material, the following four alternative monomer ratios were utilized beforehand: (a) 7:3:2, (b) 5:2:5, (c) 3:7:2, and (d) 2:5:5. A preliminary experiment was conducted where the pH was altered to match the pH of the soil, which was 7. The depth of infiltration was measured to be 3 cm. The monomer ratio was determined by increasing amounts of AM and AMPS while decreasing amounts of AA. Figure 4 demonstrates that the ratios of 5:0:7, 5:7:0, and 7:5:0 exhibit superior water absorption capabilities, but their productivity is comparatively low. On the other hand, the 5:5:2 ratio has greater productivity but lacks efficient water absorption. Hence, we selected four ratios that exhibited similar water absorption and productivity levels. The data shown in Figure 4 represent a solitary measurement conducted under specific circumstances rather than an average of several measurements. Figure 5 displays the morphology of the simulated samples after reinforcement.

The results of the water absorption and surface morphology of SAP with the four ratios show that when the ratio of each monomer is 7:3:2, SAP has the best water absorption and the least damage to the surface morphology of the earthen sites. Therefore, the 7:3:2 formula was used to strengthen the earthen sites in the follow-up site.

### 3.3. Experimental Methods

According to the water content and deterioration characteristics of the reinforcement area, combined with the indoor drip infiltration experiment, the test scheme of local reinforcement on-site is determined. Considering the permeability and feasibility of on-site reinforcement, the infiltration amount of the reinforcement agent in different water content experimental areas was calculated by Formula (1) [27,28] according to the maximum infiltration depth of 5 cm. For the area with low water content (the first floor of each tomb), the maximum saturation *S_r_* = 85%; in the area where the water content was 9~12% (the second platform of each tomb passage), the maximum saturation *S_r_* = 65% was taken; and for the high-water content area (the third floor of each tomb), the maximum saturation *S_r_* = 45%.

*U* = [1 − *ρ_d_*/(*G_s_* × *ρ_w_*)] *V* × *S_r_* (1)

where *U* is the volume of reinforcing liquid, cm^3^; *ρ_d_* is soil dry density, 1.5 g/cm^3^; *G_s_* is the proportion of site soil, which is 2.7; *ρ_w_* is the density of water, which is 1.0 g/cm^3^; *V* is the total volume of the reinforced area, cm^3^; and *S_r_* is the maximum saturation, which is 85%, 65%, and 45%.

The amount of SAP should be adjusted according to soil texture, soil particle size, regional climate characteristics, and SAP type [29]. SAP can enhance the formation of water-stable aggregates and hinder the evaporation of soil moisture. However, the effectiveness of its evaporation inhibition depends on the size of the particles. In other words, smaller particles result in better efficiency in preventing the evaporation of soil moisture [30]. Hence, it is essential to dilute the substance to establish a concentration gradient of SAP that can be compared with the experimental group. For the experiment, specific quantities of undiluted SAP-1, SAP-1.5, diluted by 1.5 times, and SAP-2, diluted by 2 times, were created per the specified criteria. A control group was established in the experiment, and the area designated as the control was sprayed with an equal volume of deionized water. Table 4 displays the level of infiltration in each region.

The spraying speed was controlled to be uniform during reinforcement, and the reinforcement material was sprayed evenly from the upper part of the test block to the test area during spraying. The spraying time interval was operated. When poor penetration occurred in the test area, the spraying was stopped. After the reinforcement material was completely penetrated, the spraying was continued after drying until the quantitative reinforcement material was sprayed.

### 3.4. Curing Methods

**Figure 6:**
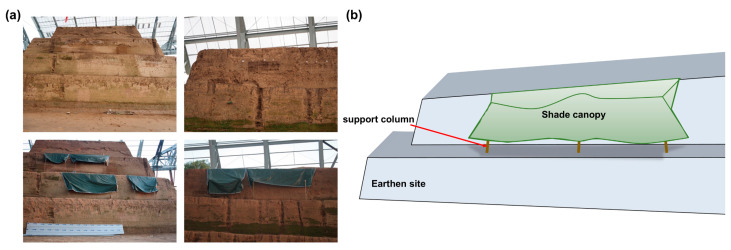
(**a**) Maintenance method of test area. (**b**) Test area awning schematic.

Outdoor studies were conducted to ensure the reinforcing agent could be uniformly distributed throughout the wall and was unaffected by natural elements such as intense sunlight and rainfall. It was essential to shield the reinforcing area from both sunlight and rainfall. Once the maintenance period of 14 days elapsed, the reinforcement area was fully exposed to the natural environment. To maintain the test area, a sunshade canopy was constructed, as seen in Figure 6. It was essential to ensure that the experimental area had enough air permeability.

The color difference compatibility, surface hardness, and macro- and micromorphology of the experimental area were studied and sampled on the 1st, 3rd, 7th, 14th, and 28th days after the completion of the reinforcement. The experiment was conducted in July. The mean daily temperature reached around 35 °C, but the relative humidity remained low at around 40%.

### 3.5. Test Method

The mineral composition of the soil at the location was studied using an X-ray diffractometer (XRD, D8 Advance, Bruker, Germany) with a scanning angle range of 5–90°.

The moisture content in the soil samples before and after reinforcement was examined using a drying technique. After the reinforcement was completed, on days 1, 3, 7, 14, and 28, a small soil sample was obtained from each experimental block's bottom right corner, sealed, and kept on site. The soil sample was weighed, and its moist weight was noted. After being dried for 24 hours at 105 °C in the oven, the soil sample was removed and returned to the lab. Weighing the bulk allowed us to assess its moisture content.

**Figure 7:**
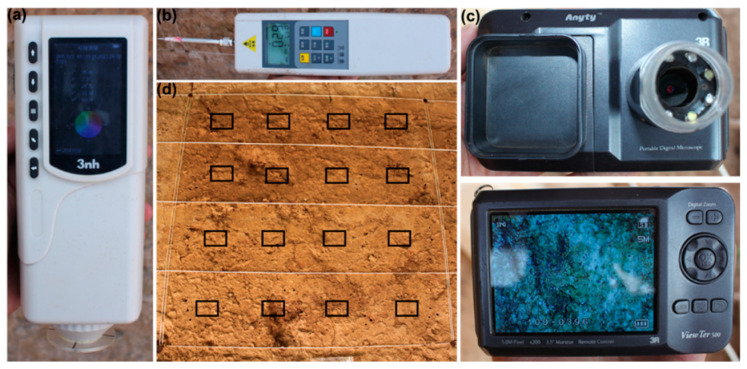
(**a**) Portable colorimeter. (**b**) Pull-out tester. (**c**) Portable microscope. (**d**) Distribution of test points.

The colorimetric study of earthen sites before and after SAP reinforcement was conducted using a portable colorimeter (model 3nh-NR60CP, Shenzhen, China) to assess changes in color differences (Figure 7a). The color difference between the earthen sites before and after reinforcement was determined using the CIE-LAB method, as described by Camerini et al. [31] and Elert et al. [32]. The calculation was based on Formula (2).

Δ*E* = [(*L*1 − *L*0)^2^ *+* (*a*1 − *a*0)^2^ + (*b*1 − *b*0)^2^]^1/2^ (2)

In Formula (2), Δ*E*: represents an overall measure of the color difference, which is often used to measure the perceived difference between two colors.

*L*1 and *L*0 represent the luminance values of the two colors, respectively. *L*1 is the brightness of the target color, and *L*0 is the brightness of the reference color.

*a*1 and *a*0 represent the values of the two colors on the red and green axes, respectively. *a*1 is the value of the target color on the red–green axis, and *a*0 is the value of the reference color on the red–green axis.

*b*1 and *b*0 represent the values of the two colors on the yellow and blue axes, respectively. *b*1 is the value of the target color on the yellow–blue axis, and *b*0 is the value of the reference color on the yellow–blue axis.

(*a*1 − *a*0), (*b*1 − *b*0), and (*L*1 − *L*0) are the differences between red and green coordinates, yellow and blue coordinates, and brightness, respectively. Δ*E* is a measure used to assess the compatibility of the reinforcement material and the reinforcement matrix regarding appearance. The compatibility improves as the value decreases. During the experiment, 16 points were randomly picked from each row on the surface of the earthen sites in the targeted region for detection, with 4 points chosen from each row. The distribution is shown in Figure 7d, and the mean value was used for analysis. When Δ*E* is less than 5, it can be considered that there is no color difference in the range of naked-eye resolution; that is, it has good compatibility with the appearance of the soil [33].

#### Surface Hardness Analysis

Surface hardness was measured by a pull-out tester (model HP-200N, HANDPI, China), as shown in Figure 7b. The probe was placed vertically into the wall to a depth of 1 cm during the measurement, and the reading was taken within 1 s. The obtained propulsion indicated the resilience of the earth. Each experimental block consisted of 16 data points sampled from the same distribution as the color difference measurement. The final strength result was obtained by averaging these data points.

Using a camera (Canon EOS 90D, Canon, Japan), the macroscopic morphology of the whole experimental area was taken, and the macroscopic morphology changes in the experimental area before and after reinforcement were recorded. Each test region served as a macroscopic indicator for quantifying the reinforcing impact of the reinforcement material in various portions.

A portable hand-held microscope (3R-View Ter-500, Beijing Aditech, Beijing, China) was used to collect the microphotographs of the surface of the reinforced area to observe the effect of SAP solidification on the surface structure of the soil (Figure 7c). The instrument was manually focused, and the lens magnification was 60 times. Each experimental region captured a minimum of 16 photographs, using the same distribution as the color difference measurement.

## 4. Results and Discussion

### 4.1. Phase Analysis

**Figure 8:**
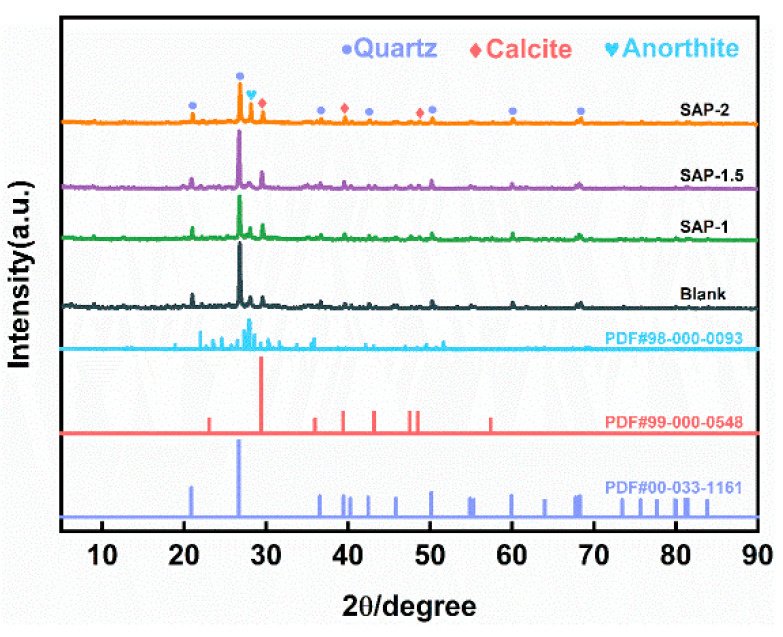
XRD of the site’s soil before and after reinforcement.

To investigate the influence of SAP reinforcement on the soil phase of the site, the soil without SAP and the soil with SAP reinforcement were examined using XRD analysis, as seen in Figure 8.

The findings indicate that including SAP does not alter the phase composition of the soil at the location, which primarily consists of quartz, calcite, and anorthite. These components are often found in soil.

### 4.2. Moisture Content

**Figure 9:**
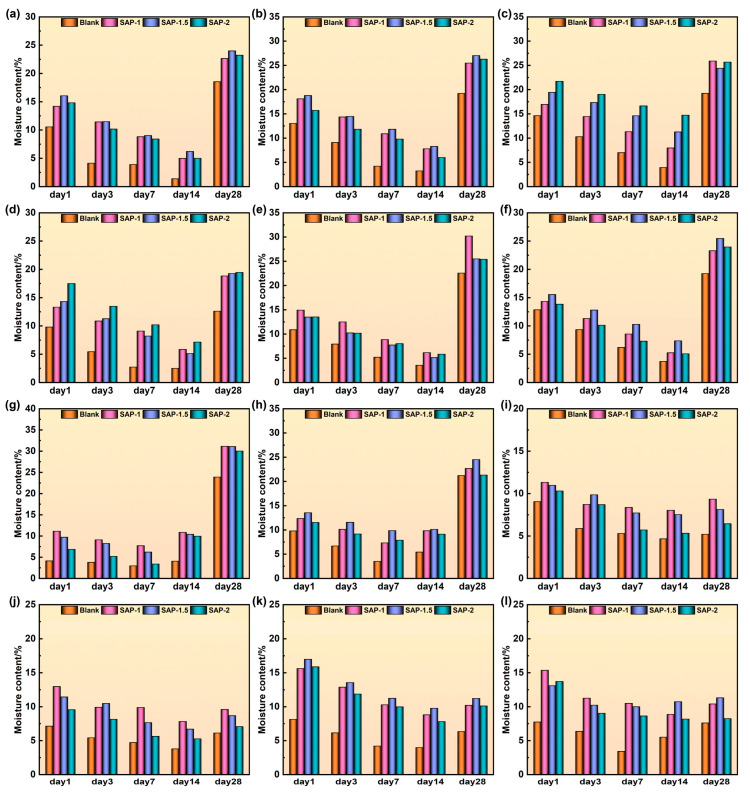
Moisture content in the experimental area. (**a**–**c**) are the first-, second-, and third-floor platforms on the east side of the North Cemetery; (**d**–**f**) are the first-, second-, and third-floor platforms on the west side of the North Cemetery; (**g**–**i**) are the first-, second-, and third-floor platforms on the north side of the East Cemetery; (**j**–**l**) are the first-, second-, and third-floor platforms on the south side of East Cemetery Road, respectively.

Figure 9 demonstrates that the soil moisture content in the region reinforced with SAP is consistently more significant than that in the non-reinforced area. This proves that adding SAP colloid may effectively enhance soil moisture levels [17].

On the 28th day, the soil surfaces were wet because of precipitation that occurred before sampling. The high porosity and extensive fissure formation of loess allow precipitation to infiltrate the soil, rapidly increasing soil saturation. Because of the increased porosity of the soil in the heavily weathered region, the presence of SAP may fill these pores, resulting in a higher water storage capacity and an increase in soil moisture content [34]. The third layer of the platform, located on the eastern and western sides of North Tomb Road and the northern side of East Tomb Road, is directly exposed to rainfall. This results in the absorption of water by SAP, significantly increasing the soil moisture content. Because of the absence of rainfall, the water content shown in Figure 9i–l remained relatively unchanged compared with the 14th day.

At each time point in 1–14 days of water loss, the soil moisture content after different concentrations of SAP reinforcement was roughly the same and was higher than that of blank soil. When the humidity of the surrounding environment decreases, SAP will slowly release water to the soil, slow the rate of soil moisture loss, enhance the soil water holding capacity [18,35], and reduce the weathering rate of the earthen sites to a certain extent [20,21].

Adding SAP can effectively reduce water evaporation, increase soil moisture, and maintain soil moisture in areas with varying degrees of weathering. Additionally, as environmental humidity decreases, SAP gradually releases water into the soil, thereby enhancing the water retention capacity of the earthen sites. The impact of wind erosion on earthen sites was mitigated.

### 4.3. Color Difference Compatibility Analysis

**Figure 10:**
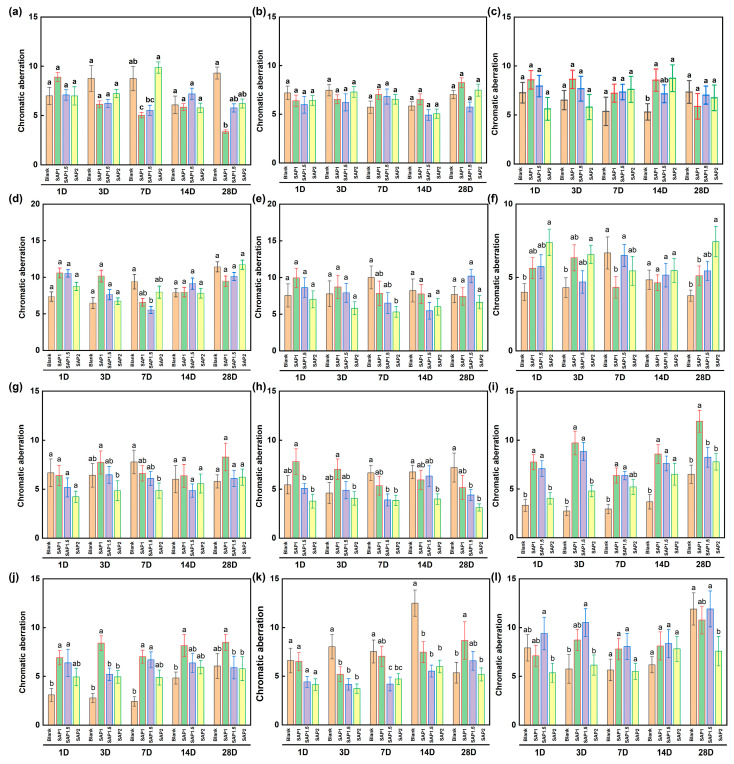
The color difference in each test area. (**a**–**c**) are the first-, second-, and third-floor platforms on the east side of the North Cemetery; (**d**–**f**) are the first-, second-, and third-floor platforms on the west side of the North Cemetery Road, respectively; (**g**–**i**) are the first-, second-, and third-floor platforms on the north side of the East Cemetery; (**j**–**l**) are the first-, second-, and third-floor platforms on the south side of East Cemetery Road, respectively. a, b, c, ab, and bc are used to represent the results of a significant comparison between different groups.

A portable chromaticity meter was used to measure the chromaticity values of the experimental area before and after reinforcement for 1, 3, 7, 14, and 28 days. The corresponding chromatic aberration values of the area before and after reinforcement were then determined for each of these days. The complexity of the soil surface at the location and the absence of human control over weather changes significantly impact the shift in chromaticity. Hence, the chromaticity test can only partially indicate the compatibility between the reinforcing agent and the soil profile. The findings must be thoroughly studied in conjunction with the macroscopic morphology. Figure 10 illustrates a clear correlation between the reduction in soil moisture content and the downward trend in the surface color difference in the earthen sites.

The reason is that with the decrease in soil moisture content, the reflection and scattering of visible light on the soil surface are strengthened, which increases the brightness of the soil surface and the color change in a lighter direction. The color of the soil changes with the change in water content. An ANOVA analysis was performed on the color difference data (*p* < 0.05), indicating that the color difference in SAP-reinforced soil was significantly different. The color difference of blank soil samples was larger than that of SAP-reinforced soil samples, and the color difference of SAP-2 reinforced area was smaller than that of SAP-1 and SAP-1.5. Therefore, SAP does not cause color difference changes other than water while maintaining soil moisture but can inhibit the color change caused by water to a certain extent.

### 4.4. Strength Analysis

**Figure 11:**
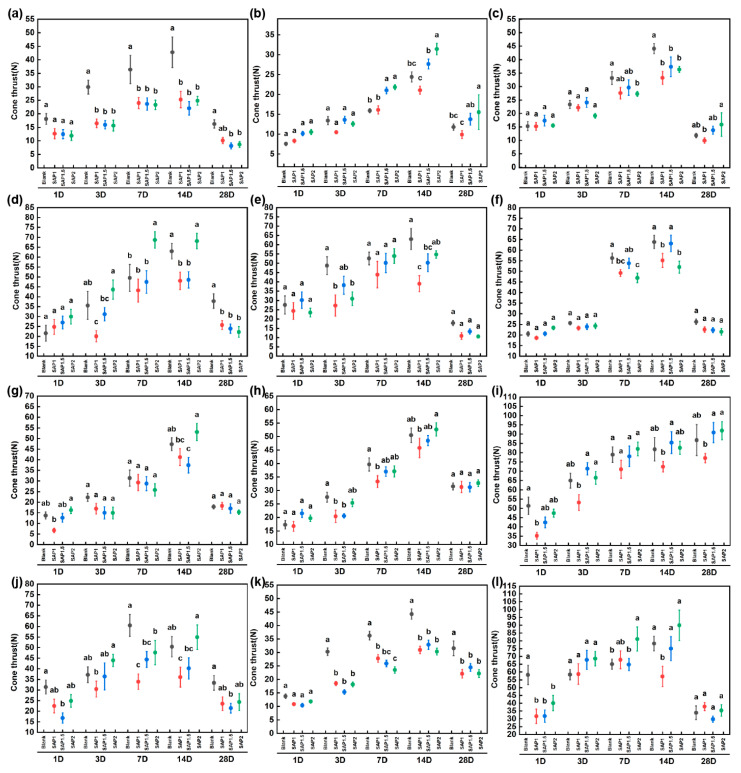
The strength of the experimental area. (**a**–**c**) are the first-, second-, and third-floor platforms on the east side of the North Cemetery; (**d**–**f**) are the first-, second-, and third-floor platforms on the west side of the North Cemetery Road, respectively; (**g**–**i**) are the first-, second-, and third-floor platforms on the north side of the East Cemetery; (**j**–**l**) are the first-, second-, and third-floor platforms on the south side of East Cemetery Road, respectively. a, b, c, ab, and bc are used to represent the results of a significant comparison between different groups.

The mechanical strength of loess is high in a dry state. Once it is soaked in water, its mechanical strength decreases sharply. It can be seen in Figure 11 that the soil strength after SAP reinforcement showed an upward trend with the decrease in water content, and the strength of each reinforcement area reached the maximum when the water content was the lowest.

At each test time point, although the strength of the SAP-reinforced area was lower than that of the blank area, the strength of the SAP-2 reinforced soil was not much different from that of the blank area. On the 28th day, under the action of rainfall, the soil moisture content continued to increase, which increased the degree of soil collapsibility deformation and softening, resulting in a decrease in the strength of the earthen sites; on the other hand, because of the increase in water content, the suction of the soil matrix will decrease rapidly, which will lead to a decrease in the strength of earthen sites [22]. The cohesion of the soil is exponentially related to the water content. As the water content gradually increases, the soil strength decreases and cohesion will tend to zero [36,37]. A total of 64 parameters from the four groups of data, i.e., blank, SAP-1, SAP-1.5, and SAP-2, were subjected to an ANOVA analysis. The results of the ANOVA showed an F-value of 17.62, which corresponded to a *p*-value of <0.001. These results indicated that there was at least one group that was significantly different from the others. Subsequently, to identify which specific groups were significantly different from each other, a multiple comparison analysis was performed, and confidence intervals for the differences in means between groups were calculated as follows:

The mean difference between blank and SAP-1 was 31.63, 95% CI [13.35, 49.90].

The mean difference between blank and SAP-1.5 was 42.69, 95% CI [23.30, 62.08].

The mean difference between blank and SAP-2 was 43.78, 95% CI [25.75, 61.82].

The mean difference between SAP-1 and SAP-1.5 was 45.20, 95% CI [35.00, 55.39].

The mean difference between SAP-1 and SAP-2 was 34.74, 95% CI [24.59, 44.88].

The mean difference between SAP-1.5 and SAP-2 was 49.10, 95% CI [36.93, 61.27].

In addition, combined reliability (CR) is a measure of the internal consistency in the ANOVA model. The CR value obtained in this study was 0.7532, which indicated that our model had an acceptable level of consistency. The ANOVA analysis (*p* < 0.05) showed that there was a significant difference in soil strength after SAP reinforcement. After SAP strengthens the soil, it will self-crosslink in situ inside the soil to form colloids, which can fill the pores between the soil to a certain extent, and it has a certain cohesion, which can agglomerate the dispersed soil particles. Hence, it improves the adhesion among the soil particles to a certain extent [38].

### 4.5. Macroscopic and Microscopic Morphology Analysis

**Figure 12:**
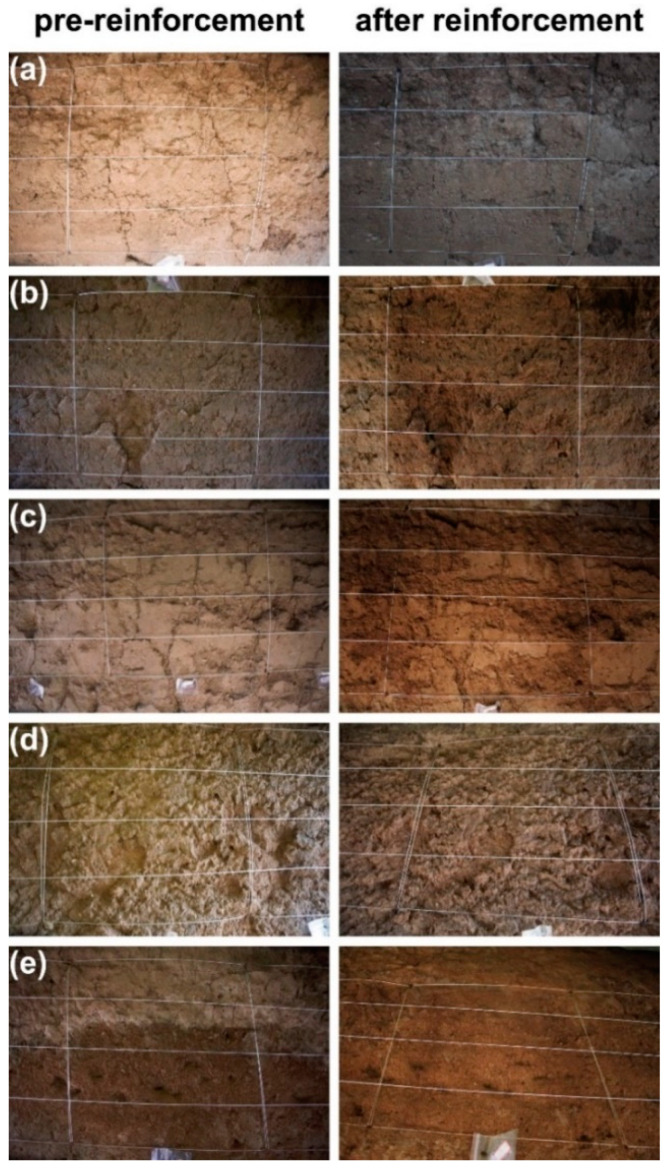
Macroscopic morphology of a typical area before and after reinforcement. (**a**) Block peeling, (**b**) fine crack, (**c**) coarse crack, (**d**) flake peeling, and (**e**) crisp powder.

Soil deterioration is often identified by erosion, fissures, and structural destabilization. This section examines the macroscopic and microscopic morphological alterations in typical soil regions before and during the implementation of SAP reinforcement. Comprehending these alterations is crucial for evaluating the efficacy of reinforcement techniques and their influence on soil health and functionality. Figure 12 displays the macroscopic morphology of each representative region both before and after reinforcement.

**Figure 13:**
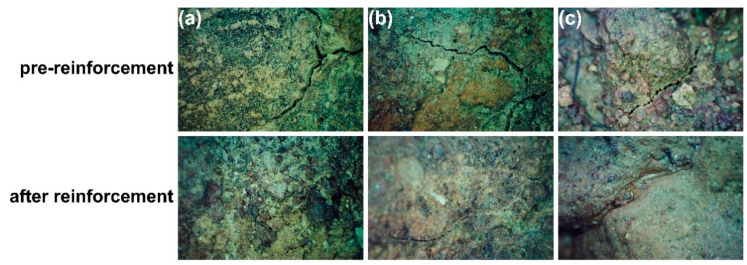
Microscopic morphology before and after crack damage reinforcement. (**a**–**c**) are fissure degradation.

Before the reinforcement of the stripping damage area, the soil was seriously eroded, the cracks were wide because of water loss and wind erosion, and the soil structure was loose and not dense (Figure 12a,d). Traditional physical reinforcement methods, such as anchors [39], can provide instant stability but may cause a certain degree of damage to the surface morphology of earthen sites [40]. After applying SAP-2, there was no visible damage to the soil surface. The number and size of surface cracks decreased, indicating improved soil moisture retention and a significant reduction in soil vulnerability to erosion. In the crack damage area (Figure 12b,c), the soil surface was full of cracks with uneven thickness before reinforcement, while the small cracks on the surface disappeared after SAP-2 reinforcement, the deeper cracks became shallow, and the cracks on the soil surface were filled microscopically (Figure 13b,c).

**Figure 14:**
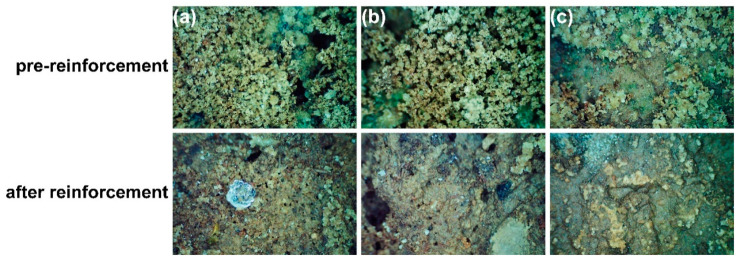
The micro-morphology of the surface of the site before and after the reinforcement of the crisp powder and salt crystallization damage. (**a**–**c**) are fragile crumble degradation.

The bonding force between particles was enhanced. This is because SAP-2 has the characteristics of water retention and moisture retention after gelation, which can increase soil moisture content and maintain soil moisture, thereby reducing the formation of cracks; the network connection and cementation between particles are the main reasons for inhibiting crack development [41]. Microbially induced calcium carbonate precipitation [42,43] can also fill cracks and enhance the adhesion between soil particles to a certain extent, improve soil strength in wind erosion resistance, and play a role in strengthening earthen sites. Still, its effect is greatly affected by environmental conditions. Figure 14a,b show that the soil particles are loose, the pores between the particles are large, and the pulverization is serious.

This phenomenon occurs because of the accumulation of salt crystals on the surface of the soil. The soluble salt undergoes a constant process of dissolution and crystallization because of ambient humidity and temperature variations. This process leads to the pulverization of the surface of the earthen sites [36]. Following reinforcement, the loose soil particles undergo compaction, resulting in increased density, and the gel becomes visible on the soil surface. SAP-2 can occupy empty spaces in loose soil and enhance the cohesiveness between soil particles, resulting in the agglomeration of scattered particles [34]. The incorporation of SAP-2 increases the soil moisture content, slows the crystallization rate of soluble salt, reduces the enrichment of salt crystallization on the soil surface [44] (Figure 14c), and hinders salt precipitation to a certain extent.

## 5. Conclusions

The addition of SAP-2 effectively decelerated the rate of water loss, elevated the soil’s water content, and effectively sustained soil moisture. This impact not only aids in preventing soil desiccation and cracking but also enhances the soil’s water retention capacity. The enhancement in soil water retention performance can also slow the crystallization rate of soluble salt, diminish the accumulation of salt crystals on the soil surface, and impede salt precipitation to a certain extent. By maintaining soil moisture, SAP-2 provided a more favorable habitat for earthen sites, contributing to preserving their original structure and characteristics. Furthermore, the incorporation of SAP-2 also significantly reduced the color difference caused by water in the earthen sites. The loss of water often leads to a change in soil color, and the addition of SAP-2 effectively stabilized the soil color so that the earthen sites can still maintain their original beauty and historical charm after the test of time. In addition to protecting the appearance characteristics of the sites, SAP-2 reduced the strength of the earthen sites to a certain extent. However, it could fill the pores between the soil to a certain extent by forming a cross-linked network with the soil with a certain cohesion. It could agglomerate dispersed soil particles, improving the adhesion between soil particles to a certain extent and making the fragile earthen sites more solid.

In summary, SAP can effectively reduce soil cracking and different degrees of flake and block spalling of soil surfaces caused by water loss in earthen sites in semi-arid areas. However, there are still some limitations. For example, its water retention and reinforcement effects may vary under different environmental conditions. Therefore, the adaptability of SAP to various climatic conditions needs to be considered to ensure its long-term effectiveness in earth site reinforcement. The depth of penetration and homogeneity in SAP may be limited by the pore structure of the earth site as well as the properties of SAP itself. Despite the limitations of SAP in earth site reinforcement, its reinforcement capacity and potential effectiveness make it promising for future earth site protection. As a polymer material, SAP has a low environmental impact in earth site reinforcement, especially in semi-arid or semi-humid areas, where it can effectively regulate soil moisture, keep the soil moist, and reduce water loss. This process not only enhances the soil structure but also promotes the bonding between soil particles, thus enhancing the compressive strength and stability of the soil.

## Figures and Tables

**Table 1:** Basic physical properties of site soil.

Natural Water Content (%)	Specific Gravity	Dry Density (g/cm^3^)	Porosity (%)	Liquid Limit (%)	Plastic Limit (%)	Plasticity Index (IP)	Organic Matter Content (g/kg)
6.4 ± 1.05	2.71	1.49 ± 0.06	35.57	34.15	14.3	19.85	16.8

**Table 2:** Element composition of site soil.

Elements	SiO_2_	Al_2_O_3_	CaO	Fe_2_O_3_	K_2_O	MgO	Na_2_O	P_2_O_5_	SO_3_	Cl
Content (%)	49.20	13.37	12.98	4.01	3.77	2.72	3.12	4.16	5.62	0.43

**Table 3:** Soluble salt anion and cation content.

Cation Content (mg/g)				Anion Content (mg/g)				Total (mg/g)
Ca^2+^	Mg^2+^	Na^+^	K^+^	NO^3−^	CO_3_^2−^	SO_4_^2−^	Cl^−^	
0.1280	0.0426	0.4415	0.0201	0.0135	0.0605	0.4237	0.0245	1.1544

**Table 4:** SAP infiltration amount of each reinforced area.

Reinforcement Area	Maximum Saturation *S_r_* (%)	Infiltration Volume (L)
The first layer platform	85	2.8
The second layer platform	65	2.1
The third layer platform	45	1.5

## Data Availability

The raw data supporting the conclusions of this article will be made available by the authors on request.

## References

[1] Li Z., Wang X., Sun M., Chen W., Guo Q., Zhang H. (2011). Conservation of Jiaohe ancient earthen site in China. J. Rock Mech. Geotech. Eng..

[2] Efthimiou N., Psomiadis E., Panagos P. (2020). Fire severity and soil erosion susceptibility mapping using multi-temporal earth observation data: The case of mati fatal wildfire in eastern attica, greece. Catena.

[3] Lan H.X., Zhao X.X., Macciotta R., Peng J.B., Li L.P., Wu Y.M., Zhu Y.B., Liu X., Zhang N., Liu S.J. (2021). The cyclic expansion and contraction characteristics of a loess slope and implications for slope stability. Sci. Rep..

[4] Wen-wu C., Na S., Guang Y. (2016). Effect of wind field on sapping quantity of earthen architecture ruins along ridge of semi-humid areas. Chin. J. Geotech. Eng..

[5] Lv J., Zhou T.H., Du Q., Wu H.H. (2017). Experimental investigation on properties of gypsum-quicklime-soil grout material in the reparation of earthen site cracks. J. Constr. Build. Mater..

[6] Martins da Silva R., Schueremans L., Oliveira D. (2009). In Grouting as a repair/strengthening solution for earth constructions. 1st WTA International PhD Symposium.

[7] Xinjun C., Chunfeng H., Jiewen Y., Yansheng G., Chenxi R. (2012). Unconfined compression strength of Tianluoshan relic soils solidified by Methyl Acrylic Acid Resin. J. Adv. Mater. Res..

[8] García-Vera V.E., Tenza-Abril A.J., Lanzón M. (2020). The effectiveness of ethyl silicate as consolidating and protective coating to extend the durability of earthen plasters. J. Constr. Build. Mater..

[9] Kong R., Zhang F., Wang G., Peng J. (2018). Stabilization of Loess Using Nano-SiO_2_. Materials.

[10] Zhang Q., Chen W., Yuan P. (2020). Experimental study on impregnation and consolidation effects of modified polyvinyl alcohol solution for coarse-grained soils: A case study on the Subashi Buddhist Temple Ruins of China. J. Bull. Eng. Geol. Environ..

[11] Lanzón M., Madrid J.A., Martínez-Arredondo A., Mónaco S. (2017). Use of diluted Ca(OH)_2_ suspensions and their transformation into nanostructured CaCO_3_ coatings: A case study in strengthening heritage materials (stucco, adobe and stone). J. Appl. Surf. Sci..

[12] García-Vera V.E., Tenza-Abril A.J., Solak A.M., Lanzón M. (2020). Calcium hydroxide nanoparticles coatings applied on cultural heritage materials: Their influence on physical characteristics of earthen plasters. J. Appl. Surf. Sci..

[13] Maravelaki-Kalaitzaki P., Kallithrakas-Kontos N., Agioutantis Z., Maurigiannakis S., Korakaki D. (2008). A comparative study of porous limestones treated with silicon-based strengthening agents. Prog. Org. Coat..

[14] Kim E.K., Won J., Do J.Y., Kim S.D., Kang Y.S. (2009). Effects of silica nanoparticle and GPTMS addition on TEOS-based stone consolidants. J. Cult. Herit..

[15] Biscontin G., Maravelaki P., Zendri E., Glisenti A. (1992). Siliconic and acrylic resins dispersed in water as protectives for stone surface. J. MRS Online Proc. Libr..

[16] Carretti E., Dei L. (2004). Physicochemical characterization of acrylic polymeric resins coating porous materials of artistic interest. J. Prog. Org. Coat..

[17] Zhang F., Li J., Lei Y., Luo P. (2010). Effects of super absorbent polymer on retention properties of soil water and nutrient. J. Basic Sci. Eng..

[18] Yazdani F., Allahdadi I., Akbari G.A. (2007). Impact of superabsorbent polymer on yield and growth analysis of soybean (*Glycine max* L.) under drought stress condition. Pak. J. Biol. Sci..

[19] Bai W., Zhang H., Liu B., Wu Y., Song J. (2010). Effects of super-absorbent polymers on the physical and chemical properties of soil following different wetting and drying cycles. Soil Use Manag..

[20] Zheng T., Liang Y.H., Ye S.H., He Z.Y. (2009). Superabsorbent hydrogels as carriers for the controlled-release of urea: Experiments and a mathematical model describing the release rate. Biosyst. Eng..

[21] Cao Y.B., Wang B.T., Guo H.Y., Xiao H.J., Wei T.T. (2017). The effect of super absorbent polymers on soil and water conservation on the terraces of the loess plateau. Ecol. Eng..

[22] Wu S., Wu P., Feng H., Bu C. (2010). Influence of amendments on soil structure and soil loss under simulated rainfall China’s loess plateau. Afr. J. Biotechnol..

[23] (2022). Agricultural and Forestry Absorbent Polymer.

[24] (2016). Soil Conditioner Evaluation General Requirements for Effect Testing and Assessment.

[25] Lin Y.T., Mao W.J., Deng K., Kang H.Y., Shi M.Y., Sun M.L. (2024). Interpreting the initial growth process of surface peeling: Microstructural evolutions of soil deterioration on archaeological sites. Int. J. Archit. Herit..

[26] (2019). Standard for Geotechnical Testing Method.

[27] Chen W.W., Zhang Y.M., Zhang J.K., Dai P.F. (2018). Consolidation effect of composite materials on earthen sites. Constr. Build. Mater..

[28] Zhao D., Lu W., Wang Y., Mao X., Ai Y., Jiang H. (2016). Experimental studies on earthen architecture sites consolidated with BS materials in arid regions. J. Adv. Mater. Sci. Eng..

[29] Kong W., Li Q., Li X., Su Y., Yue Q., Gao B. (2019). A biodegradable biomass-based polymeric composite for slow release and water retention. J. Environ. Manag..

[30] Zhao W.J., Cao T.H., Dou P.X., Sheng J., Luo M.Q. (2019). Effect of various concentrations of superabsorbent polymers on soil particle-size distribution and evaporation with sand mulching. Sci. Rep..

[31] Camerini R., Chelazzi D., Giorgi R., Baglioni P. (2019). Hybrid nano-composites for the consolidation of earthen masonry. J. Colloid Interface Sci..

[32] Elert K., Pardo E.S., Rodriguez-Navarro C. (2015). Alkaline activation as an alternative method for the consolidation of earthen architecture. J. Cult. Herit..

[33] Rodrigues J.D., Grossi A. (2007). Indicators and ratings for the compatibility assessment of conservation actions. J. Cult. Herit..

[34] Bahmani M., Noorzad A., Hamedi J., Sali F. (2017). The role of bacillus pasteurii on the change of parameters of sands according to temperatur compresion and wind erosion resistance. J. CleanWAS.

[35] Yang Q.Y., Li C.W. (2012). Research on the Impact of Drying and Wetting Cycle of Capillary Water on Weathering of Soil Sites. Chin. J. Undergr. Space Eng..

[36] Zhang Y., Zhong X., Lin J., Zhao D., Jiang F., Wang M.-K., Ge H., Huang Y. (2020). Effects of fractal dimension and water content on the shear strength of red soil in the hilly granitic region of southern China. J. Geomorphol..

[37] Gerard C. (1965). The influence of soil moisture, soil texture, drying conditions, and exchangeable cations on soil strength. Soil Sci. Soc. Am. J..

[38] Matsushi Y., Matsukura Y. (2006). Cohesion of unsaturated residual soils as a function of volumetric water content. J. Bull. Eng. Geol. Environ..

[39] Bian X., Cao Y.P., Wang Z.F., Ding G.Q., Lei G.H. (2017). Effect of super-absorbent polymer on the undrained shear behavior of cemented dredged clay with high water content. J. Mater. Civ. Eng..

[40] Zhang J., Chen W., He F., Li Z., Sun M. (2014). Field experimental study on anchorage perfromance of gfrp at conservation earthen sites. J. Eng. Geol..

[41] Yulan W., Jian G., Weixi Z., Fan L. (2023). Experimental research on the performance of a novel geo-filament anchor for an earthen architectural site. J. Herit. Sci..

[42] Zhang Q., Chen W., Han N., Cai T., Du Y. (2021). Feasibility of polyvinyl alcohol-treated soil in a mud state as the anti-weathering material for earthen sites. Int. J. Archit. Herit..

[43] Liu J., Li G., Li X.A. (2021). Geotechnical engineering properties of soils solidified by microbially induced CaCO_3_ precipitation (MICP). Adv. Civ. Eng..

[44] Shu M., Yu Y., Yin M., Wang J., Bandala E.R., Rodrigo-Comino J. (2024). Super absorbent polymer (sap) on water-salt transport in saline alkali soil: Effects of dosage, height and thickness. Eur. J. Soil Sci..

